# 2-Phenyl-3-(phenyl­sulfin­yl)naphtho[1,2-*b*]furan

**DOI:** 10.1107/S1600536809023101

**Published:** 2009-06-20

**Authors:** Hong Dae Choi, Pil Ja Seo, Byeng Wha Son, Uk Lee

**Affiliations:** a Department of Chemistry, Dongeui University, San 24 Kaya-dong Busanjin-gu, Busan 614-714, Republic of Korea; b Department of Chemistry, Pukyong National University, 599-1 Daeyeon 3-dong, Nam-gu, Busan 608-737, Republic of Korea

## Abstract

In the title compound, C_24_H_16_O_2_S, the O atom and the phenyl group of the phenyl­sulfinyl substituent lie on opposite sides of the plane of the naphthofuran fragment; the phenyl ring is almost perpendicular to this plane [81.54 (5)°]. The 2-phenyl ring is rotated out of the naphthofuran plane, making a dihedral angle of 18.2 (1)°.

## Related literature

For the crystal structures of similar naphtho[1,2-*b*]furan derivatives, see: Choi *et al.* (2008**a* ▶,b* ▶). For the biological and pharmacological activity of naphthofuran compounds, see: Goel & Dixit (2004 ▶); Piloto *et al.* (2005 ▶).
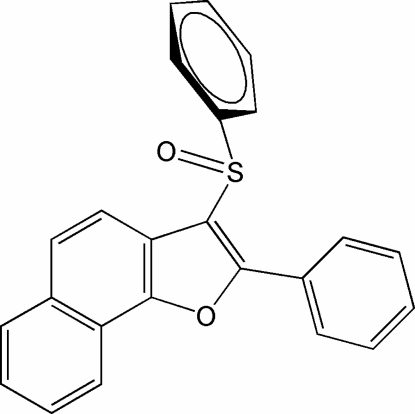


## Experimental

### 

#### Crystal data

C_24_H_16_O_2_S
*M* *_r_* = 368.44
Monoclinic, 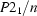

*a* = 10.123 (1) Å
*b* = 14.109 (2) Å
*c* = 12.410 (2) Å
β = 99.743 (2)°
*V* = 1746.9 (4) Å^3^
*Z* = 4
Mo *K*α radiation
μ = 0.20 mm^−1^
*T* = 173 K
0.20 × 0.20 × 0.15 mm

#### Data collection

Bruker SMART CCD diffractometer
Absorption correction: none
10523 measured reflections
3804 independent reflections
2555 reflections with *I* > 2σ(*I*)
*R*_int_ = 0.079

#### Refinement

*R*[*F* ^2^ > 2σ(*F* ^2^)] = 0.053
*wR*(*F* ^2^) = 0.137
*S* = 1.05
3804 reflections
244 parameters
H-atom parameters constrained
Δρ_max_ = 0.41 e Å^−3^
Δρ_min_ = −0.41 e Å^−3^

### 

Data collection: *SMART* (Bruker, 2001 ▶); cell refinement: *SAINT* (Bruker, 2001 ▶); data reduction: *SAINT*; program(s) used to solve structure: *SHELXS97* (Sheldrick, 2008 ▶); program(s) used to refine structure: *SHELXL97* (Sheldrick, 2008 ▶); molecular graphics: *ORTEP-3* (Farrugia, 1997 ▶); software used to prepare material for publication: *SHELXL97*.

## Supplementary Material

Crystal structure: contains datablocks global, I. DOI: 10.1107/S1600536809023101/hg2520sup1.cif

Structure factors: contains datablocks I. DOI: 10.1107/S1600536809023101/hg2520Isup2.hkl

Additional supplementary materials:  crystallographic information; 3D view; checkCIF report

## Figures and Tables

**Fig. 1.**
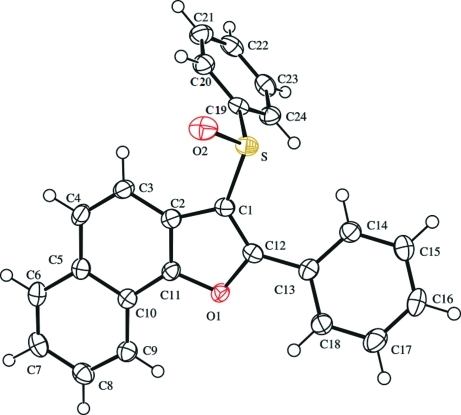
The molecular structure of the title compound with the atom numbering scheme. Displacement ellipsoids are drawn at the 50% probability level. H atoms are presented as a small cycles of arbitrary radius.

## supplementary crystallographic information

### Comment

The naphthofuran compounds have attracted widespread interest in view of their biological and pharmacological activities (Goel & Dixit, 2004; Piloto *et al.*, 2005). This work is related to our communications on the synthesis and structures of naphtho[1,2-b]furan analogues, *viz*. 2-methyl-3-(phenylsulfonyl)naphtho[1,2-b]furan (Choi *et al.*,  2008*a*) and 3-(4-chlorophenylsulfonyl)-2-methylnaphtho[1,2-b]furan (Choi *et al.*, 2008*b*). We report the crystal structure of the title compound (I), 2-phenyl-3-(phenylsulfinyl)naphtho[1,2-b]furan (Fig. 1).

The naphthofuran unit is essentially planar, with a mean deviation of 0.023 (2) Å from the least-squares plane defined by the thirteen constituent atoms. The dihedral angle in (I) formed by the plane of the naphthofuran ring and the plane of 2-phenyl ring is 18.2 (1)°, and the phenyl ring (C19-C24) with 81.54 (5)° lies toward the naphthofuran plane.

### Experimental

The 77% 3-chloroperoxybenzoic acid (77%, 179 mg, 0.8 mmol) was added in small portions to a stirred solution of 2-phenyl-3-(phenylsulfanyl)naphtho[1,2-b]furan (282 mg, 0.8 mmol) in dichloromethane (30 mL) at 273 K. After being stirred at room temperature for 3h, the mixture was washed with saturated sodium bicarbonate solution and the organic layer was separated, dried over magnesium sulfate, filtered and concentrated in vacuum. The residue was purified by column chromatography (hexane-ethyl acetate, 2 : 1 v/v) to afford the title compound as a colorless solid [yield 80%, m.p. 472-473 K; R_f_ = 0.52 (hexane-ethyl acetate, 2:1 v/v)]. Single crystals suitable for X-ray diffraction were prepared by slow evaporation of a solution of the title compound in benzene at room temperature.

### Refinement

All H atoms were positioned geometrically and refined using a riding model, with C–H = 0.95 Å for aromatic H atoms and with Uiso(H) = 1.2Ueq(C) for aromatic H atoms.

### Crystal data

**Table:**

C_24_H_16_O_2_S	*F*(000) = 768
*M**_r_* = 368.44	*D*_x_ = 1.401 Mg m^−^^3^
Monoclinic, *P*2_1_/*n*	Mo *K*α radiation, λ = 0.71073 Å
Hall symbol: -P 2yn	Cell parameters from 2794 reflections
*a* = 10.123 (1) Å	θ = 2.4–28.0°
*b* = 14.109 (2) Å	µ = 0.20 mm^−^^1^
*c* = 12.410 (2) Å	*T* = 173 K
β = 99.743 (2)°	Block, colorless
*V* = 1746.9 (4) Å^3^	0.20 × 0.20 × 0.15 mm
*Z* = 4	

### Data collection

**Table:**

Bruker SMART CCD diffractometer	2555 reflections with *I* > 2σ(*I*)
Radiation source: fine-focus sealed tube	*R*_int_ = 0.079
graphite	θ_max_ = 27.0°, θ_min_ = 2.2°
Detector resolution: 10.0 pixels mm^-1^	*h* = −12→12
φ and ω scans	*k* = −18→17
10523 measured reflections	*l* = −15→10
3804 independent reflections	

### Refinement

**Table:**

Refinement on *F*^2^	Primary atom site location: structure-invariant direct methods
Least-squares matrix: full	Secondary atom site location: difference Fourier map
*R*[*F*^2^ > 2σ(*F*^2^)] = 0.053	Hydrogen site location: difference Fourier map
*wR*(*F*^2^) = 0.137	H-atom parameters constrained
*S* = 1.05	*w* = 1/[σ^2^(*F*_o_^2^) + (0.0554*P*)^2^ + 0.5717*P*] where *P* = (*F*_o_^2^ + 2*F*_c_^2^)/3
3804 reflections	(Δ/σ)_max_ < 0.001
244 parameters	Δρ_max_ = 0.41 e Å^−^^3^
0 restraints	Δρ_min_ = −0.41 e Å^−^^3^

### Special details

**Table:**

Geometry. All esds (except the esd in the dihedral angle between two l.s. planes) are estimated using the full covariance matrix. The cell esds are taken into account individually in the estimation of esds in distances, angles and torsion angles; correlations between esds in cell parameters are only used when they are defined by crystal symmetry. An approximate (isotropic) treatment of cell esds is used for estimating esds involving l.s. planes.
Refinement. Refinement of F^2^ against ALL reflections. The weighted R-factor wR and goodness of fit S are based on F^2^, conventional R-factors R are based on F, with F set to zero for negative F^2^. The threshold expression of F^2^ > 2sigma(F^2^) is used only for calculating R-factors(gt) etc. and is not relevant to the choice of reflections for refinement. R-factors based on F^2^ are statistically about twice as large as those based on F, and R- factors based on ALL data will be even larger.

### Fractional atomic coordinates and isotropic or equivalent isotropic displacement parameters (Å^2^)

**Table:**

	*x*	*y*	*z*	*U*_iso_*/*U*_eq_	
S	0.28091 (6)	0.00804 (5)	0.16729 (5)	0.02695 (19)	
O1	0.40566 (16)	0.15573 (12)	0.43697 (13)	0.0242 (4)	
O2	0.20908 (19)	0.07138 (13)	0.08044 (15)	0.0371 (5)	
C1	0.3042 (2)	0.07384 (17)	0.2906 (2)	0.0237 (5)	
C2	0.2072 (2)	0.13705 (17)	0.3244 (2)	0.0245 (5)	
C3	0.0698 (2)	0.15748 (18)	0.2865 (2)	0.0287 (6)	
H3	0.0206	0.1244	0.2261	0.034*	
C4	0.0109 (2)	0.22564 (19)	0.3393 (2)	0.0296 (6)	
H4	−0.0810	0.2396	0.3146	0.035*	
C5	0.0812 (2)	0.27738 (18)	0.4303 (2)	0.0257 (6)	
C6	0.0202 (3)	0.34960 (19)	0.4835 (2)	0.0306 (6)	
H6	−0.0720	0.3636	0.4600	0.037*	
C7	0.0913 (3)	0.39968 (19)	0.5680 (2)	0.0331 (6)	
H7	0.0481	0.4480	0.6025	0.040*	
C8	0.2281 (3)	0.38060 (19)	0.6046 (2)	0.0322 (6)	
H8	0.2768	0.4163	0.6631	0.039*	
C9	0.2907 (3)	0.31082 (18)	0.5560 (2)	0.0287 (6)	
H9	0.3829	0.2980	0.5812	0.034*	
C19	0.1603 (2)	−0.07478 (17)	0.2024 (2)	0.0247 (5)	
C10	0.2198 (2)	0.25777 (17)	0.4690 (2)	0.0234 (5)	
C11	0.2748 (2)	0.18564 (17)	0.4126 (2)	0.0236 (5)	
C12	0.4218 (2)	0.08750 (17)	0.3604 (2)	0.0238 (5)	
C13	0.5573 (2)	0.04856 (18)	0.3710 (2)	0.0245 (6)	
C14	0.5820 (3)	−0.03834 (18)	0.3242 (2)	0.0291 (6)	
H14	0.5092	−0.0741	0.2861	0.035*	
C15	0.7112 (3)	−0.07276 (19)	0.3326 (2)	0.0335 (6)	
H15	0.7268	−0.1318	0.3001	0.040*	
C16	0.8175 (3)	−0.0217 (2)	0.3881 (2)	0.0359 (7)	
H16	0.9064	−0.0449	0.3927	0.043*	
C17	0.7944 (3)	0.0634 (2)	0.4371 (2)	0.0372 (7)	
H17	0.8673	0.0976	0.4773	0.045*	
C18	0.6658 (3)	0.0989 (2)	0.4279 (2)	0.0311 (6)	
H18	0.6512	0.1580	0.4605	0.037*	
C20	0.0331 (3)	−0.07786 (19)	0.1411 (2)	0.0308 (6)	
H20	0.0073	−0.0336	0.0837	0.037*	
C21	−0.0565 (3)	−0.1460 (2)	0.1641 (2)	0.0349 (7)	
H21	−0.1441	−0.1486	0.1222	0.042*	
C22	−0.0190 (3)	−0.21003 (19)	0.2474 (2)	0.0351 (7)	
H22	−0.0813	−0.2560	0.2636	0.042*	
C23	0.1093 (3)	−0.20754 (19)	0.3078 (2)	0.0318 (6)	
H23	0.1350	−0.2521	0.3649	0.038*	
C24	0.1998 (3)	−0.14042 (18)	0.2852 (2)	0.0290 (6)	
H24	0.2882	−0.1390	0.3257	0.035*	

### Atomic displacement parameters (Å^2^)

**Table:**

	*U*^11^	*U*^22^	*U*^33^	*U*^12^	*U*^13^	*U*^23^
S	0.0311 (3)	0.0275 (4)	0.0222 (3)	−0.0035 (3)	0.0044 (3)	−0.0006 (3)
O1	0.0217 (8)	0.0279 (10)	0.0219 (9)	0.0022 (7)	0.0003 (7)	−0.0001 (7)
O2	0.0493 (12)	0.0350 (11)	0.0250 (10)	−0.0060 (9)	0.0006 (9)	0.0065 (8)
C1	0.0273 (13)	0.0217 (13)	0.0215 (13)	−0.0017 (10)	0.0021 (10)	0.0016 (10)
C2	0.0269 (13)	0.0220 (13)	0.0238 (13)	−0.0018 (10)	0.0025 (11)	0.0047 (11)
C3	0.0259 (13)	0.0286 (14)	0.0291 (14)	−0.0024 (11)	−0.0027 (11)	0.0000 (12)
C4	0.0203 (12)	0.0316 (15)	0.0350 (15)	0.0024 (11)	−0.0007 (11)	0.0048 (12)
C5	0.0274 (13)	0.0259 (14)	0.0242 (13)	0.0018 (11)	0.0056 (11)	0.0062 (11)
C6	0.0280 (13)	0.0300 (15)	0.0350 (16)	0.0059 (11)	0.0087 (12)	0.0084 (12)
C7	0.0413 (15)	0.0288 (15)	0.0320 (15)	0.0071 (12)	0.0144 (13)	0.0011 (12)
C8	0.0397 (15)	0.0289 (15)	0.0278 (14)	0.0013 (12)	0.0056 (12)	−0.0007 (12)
C9	0.0290 (13)	0.0302 (15)	0.0264 (14)	0.0015 (11)	0.0038 (11)	0.0019 (11)
C19	0.0303 (13)	0.0212 (13)	0.0233 (13)	−0.0026 (11)	0.0061 (11)	−0.0048 (10)
C10	0.0253 (13)	0.0239 (13)	0.0216 (13)	−0.0006 (10)	0.0053 (10)	0.0046 (10)
C11	0.0221 (12)	0.0231 (13)	0.0246 (13)	0.0001 (10)	0.0011 (10)	0.0048 (10)
C12	0.0299 (13)	0.0209 (13)	0.0210 (13)	−0.0023 (11)	0.0056 (10)	0.0008 (10)
C13	0.0254 (12)	0.0293 (14)	0.0188 (12)	0.0019 (10)	0.0032 (10)	0.0054 (11)
C14	0.0288 (13)	0.0268 (14)	0.0313 (15)	−0.0017 (11)	0.0041 (12)	0.0022 (12)
C15	0.0359 (15)	0.0286 (15)	0.0365 (16)	0.0080 (12)	0.0077 (13)	0.0038 (13)
C16	0.0273 (14)	0.0436 (17)	0.0366 (16)	0.0120 (13)	0.0048 (12)	0.0081 (14)
C17	0.0300 (14)	0.0487 (19)	0.0302 (15)	0.0029 (13)	−0.0024 (12)	−0.0006 (13)
C18	0.0313 (14)	0.0335 (16)	0.0266 (14)	0.0035 (12)	−0.0006 (11)	−0.0020 (12)
C20	0.0333 (14)	0.0303 (15)	0.0277 (14)	0.0002 (12)	0.0017 (12)	−0.0001 (12)
C21	0.0285 (14)	0.0397 (17)	0.0363 (16)	−0.0065 (12)	0.0048 (12)	−0.0060 (13)
C22	0.0410 (16)	0.0260 (15)	0.0417 (17)	−0.0080 (12)	0.0167 (14)	−0.0075 (13)
C23	0.0446 (16)	0.0229 (14)	0.0294 (15)	0.0025 (12)	0.0103 (13)	0.0031 (11)
C24	0.0317 (14)	0.0298 (14)	0.0243 (13)	−0.0015 (11)	0.0011 (11)	−0.0007 (11)

### Geometric parameters (Å, °)

**Table:**

S—O2	1.4912 (19)	C19—C24	1.390 (3)
S—C1	1.771 (3)	C10—C11	1.403 (3)
S—C19	1.796 (3)	C12—C13	1.463 (3)
O1—C11	1.375 (3)	C13—C18	1.395 (3)
O1—C12	1.381 (3)	C13—C14	1.397 (4)
C1—C12	1.363 (3)	C14—C15	1.383 (4)
C1—C2	1.440 (3)	C14—H14	0.9500
C2—C11	1.372 (3)	C15—C16	1.378 (4)
C2—C3	1.420 (3)	C15—H15	0.9500
C3—C4	1.356 (4)	C16—C17	1.384 (4)
C3—H3	0.9500	C16—H16	0.9500
C4—C5	1.429 (4)	C17—C18	1.381 (4)
C4—H4	0.9500	C17—H17	0.9500
C5—C6	1.412 (4)	C18—H18	0.9500
C5—C10	1.431 (3)	C20—C21	1.384 (4)
C6—C7	1.365 (4)	C20—H20	0.9500
C6—H6	0.9500	C21—C22	1.377 (4)
C7—C8	1.408 (4)	C21—H21	0.9500
C7—H7	0.9500	C22—C23	1.386 (4)
C8—C9	1.365 (4)	C22—H22	0.9500
C8—H8	0.9500	C23—C24	1.379 (4)
C9—C10	1.407 (3)	C23—H23	0.9500
C9—H9	0.9500	C24—H24	0.9500
C19—C20	1.381 (3)		
			
O2—S—C1	106.72 (11)	C2—C11—C10	125.0 (2)
O2—S—C19	107.27 (11)	O1—C11—C10	124.2 (2)
C1—S—C19	97.33 (11)	C1—C12—O1	110.1 (2)
C11—O1—C12	106.53 (18)	C1—C12—C13	135.2 (2)
C12—C1—C2	107.0 (2)	O1—C12—C13	114.7 (2)
C12—C1—S	126.7 (2)	C18—C13—C14	118.4 (2)
C2—C1—S	125.37 (18)	C18—C13—C12	120.0 (2)
C11—C2—C3	119.4 (2)	C14—C13—C12	121.5 (2)
C11—C2—C1	105.6 (2)	C15—C14—C13	120.7 (2)
C3—C2—C1	135.0 (2)	C15—C14—H14	119.7
C4—C3—C2	118.0 (2)	C13—C14—H14	119.7
C4—C3—H3	121.0	C16—C15—C14	120.2 (3)
C2—C3—H3	121.0	C16—C15—H15	119.9
C3—C4—C5	122.9 (2)	C14—C15—H15	119.9
C3—C4—H4	118.6	C15—C16—C17	119.8 (2)
C5—C4—H4	118.6	C15—C16—H16	120.1
C6—C5—C4	122.7 (2)	C17—C16—H16	120.1
C6—C5—C10	117.6 (2)	C18—C17—C16	120.3 (3)
C4—C5—C10	119.7 (2)	C18—C17—H17	119.8
C7—C6—C5	121.1 (2)	C16—C17—H17	119.8
C7—C6—H6	119.4	C17—C18—C13	120.5 (3)
C5—C6—H6	119.4	C17—C18—H18	119.7
C6—C7—C8	120.7 (3)	C13—C18—H18	119.7
C6—C7—H7	119.6	C19—C20—C21	119.4 (3)
C8—C7—H7	119.6	C19—C20—H20	120.3
C9—C8—C7	120.0 (3)	C21—C20—H20	120.3
C9—C8—H8	120.0	C22—C21—C20	120.2 (3)
C7—C8—H8	120.0	C22—C21—H21	119.9
C8—C9—C10	120.5 (2)	C20—C21—H21	119.9
C8—C9—H9	119.8	C21—C22—C23	120.2 (3)
C10—C9—H9	119.8	C21—C22—H22	119.9
C20—C19—C24	120.7 (2)	C23—C22—H22	119.9
C20—C19—S	119.7 (2)	C24—C23—C22	120.1 (3)
C24—C19—S	119.35 (19)	C24—C23—H23	120.0
C11—C10—C9	125.1 (2)	C22—C23—H23	120.0
C11—C10—C5	115.0 (2)	C23—C24—C19	119.3 (2)
C9—C10—C5	120.0 (2)	C23—C24—H24	120.3
C2—C11—O1	110.7 (2)	C19—C24—H24	120.3
			
O2—S—C1—C12	−127.7 (2)	C12—O1—C11—C2	1.2 (3)
C19—S—C1—C12	121.7 (2)	C12—O1—C11—C10	−177.3 (2)
O2—S—C1—C2	39.8 (2)	C9—C10—C11—C2	−176.7 (2)
C19—S—C1—C2	−70.8 (2)	C5—C10—C11—C2	1.9 (4)
C12—C1—C2—C11	0.7 (3)	C9—C10—C11—O1	1.6 (4)
S—C1—C2—C11	−168.88 (19)	C5—C10—C11—O1	−179.8 (2)
C12—C1—C2—C3	179.5 (3)	C2—C1—C12—O1	0.0 (3)
S—C1—C2—C3	10.0 (4)	S—C1—C12—O1	169.41 (17)
C11—C2—C3—C4	0.6 (4)	C2—C1—C12—C13	−178.7 (3)
C1—C2—C3—C4	−178.1 (3)	S—C1—C12—C13	−9.4 (4)
C2—C3—C4—C5	0.2 (4)	C11—O1—C12—C1	−0.7 (3)
C3—C4—C5—C6	178.7 (2)	C11—O1—C12—C13	178.3 (2)
C3—C4—C5—C10	0.0 (4)	C1—C12—C13—C18	158.3 (3)
C4—C5—C6—C7	−177.9 (2)	O1—C12—C13—C18	−20.4 (3)
C10—C5—C6—C7	0.8 (4)	C1—C12—C13—C14	−20.9 (4)
C5—C6—C7—C8	0.0 (4)	O1—C12—C13—C14	160.3 (2)
C6—C7—C8—C9	−0.5 (4)	C18—C13—C14—C15	−1.0 (4)
C7—C8—C9—C10	0.3 (4)	C12—C13—C14—C15	178.3 (2)
O2—S—C19—C20	7.9 (2)	C13—C14—C15—C16	0.3 (4)
C1—S—C19—C20	118.0 (2)	C14—C15—C16—C17	1.2 (4)
O2—S—C19—C24	−177.6 (2)	C15—C16—C17—C18	−2.0 (4)
C1—S—C19—C24	−67.5 (2)	C16—C17—C18—C13	1.3 (4)
C8—C9—C10—C11	179.1 (2)	C14—C13—C18—C17	0.2 (4)
C8—C9—C10—C5	0.5 (4)	C12—C13—C18—C17	−179.1 (2)
C6—C5—C10—C11	−179.7 (2)	C24—C19—C20—C21	1.3 (4)
C4—C5—C10—C11	−1.0 (3)	S—C19—C20—C21	175.7 (2)
C6—C5—C10—C9	−1.0 (4)	C19—C20—C21—C22	0.1 (4)
C4—C5—C10—C9	177.7 (2)	C20—C21—C22—C23	−1.0 (4)
C3—C2—C11—O1	179.8 (2)	C21—C22—C23—C24	0.5 (4)
C1—C2—C11—O1	−1.2 (3)	C22—C23—C24—C19	0.9 (4)
C3—C2—C11—C10	−1.8 (4)	C20—C19—C24—C23	−1.8 (4)
C1—C2—C11—C10	177.3 (2)	S—C19—C24—C23	−176.2 (2)
